# Supplementary material for: Economic complexity of prefectures in Japan
Source: PLoS One. 2020 Aug 27;15(8):e0238017. doi: 10.1371/journal.pone.0238017 (PMC7451641; doi:10.1371/journal.pone.0238017)
Supplement: S1 Appendix — (PDF) [file pone.0238017.s001.pdf]

S1 Appendix: Economic complexity of prefectures in Japan

- S1 Table represents the code of the prefectures and the number of firms in the 47 prefectures in Japan.
- S1 Fig shows the 8 regions and 47 prefectures in Japan.
- S2 Table and S3 Table represent the industrial sectors and their divisions.
- S4 Table compare the ranking of prefecture by economic complexity index method and fitness complexity method.
- S5 Table is list of the top and bottom 10 industrial sectors ranked by economic complexity index method and fitness complexity method.
- Section "the average prefectural economic complexity of regions in Japan" relate the obtained ECI with the regions in Japan
- S2 Fig shows the variation of average prefectural economic coplexity index with average gross prefectural product per capita and average prefectural income per person in eight regions and Tokyo.

S1 Table. **Prefectures, regions and the firm distribution**

ID	Code	Prefecture	Region	# Firms
1	HK	Hokkaido	Hokkaido	53497
2	AO	Aomori		13811
3	IW	Iwate		11213
4	MG	Miyagi	Tohoku	20443
5	AK	Akita		10795
6	YT	Yamagata		11970
7	FS	Fukushima		17534
8	IB	Ibaraki		22628
9	TC	Tochigi		16751
10	GM	Gunma		18669
11	ST	Saitama	Kanto	39722
12	CH	Chiba		34072
13	TK	Tokyo		134479
14	KN	Kanagawa		51245
15	NI	Niigata		22945
16	TY	Toyama		11988
17	IS	Ishikawa		10577
18	FI	Fukui		10257
19	YN	Yamanashi	Chubu	9214
20	NN	Nagano		19361
21	GF	Gifu		17538
22	SZ	Shizuoka		31461
23	AI	Aichi		59070
24	ME	Mie		15523
25	SH	Shiga		9224
26	KY	Kyoto		19616
27	OS	Osaka	Kansai	69735
28	HG	Hyogo		33550
29	NR	Nara		8197
30	WK	Wakayama		8363
31	TT	Tottori		5045
32	SM	Shimane		7213
33	OY	Okayama	Chugoku	17663
34	HS	Hiroshima		26578
35	YC	Yamaguchi		11173
36	TS	Tokushima		7796
37	KG	Kagawa	Shikoku	10676
38	EH	Ehime		13826
39	KC	Kochi		7102
40	FO	Fukuoka		38274
41	SG	Saga		7162
42	NS	Nagasaki		10413
43	KM	Kumamoto	Kyushu	13687
44	OT	Oita		10713
45	MZ	Miyazaki		10242
46	KS	Kagoshima		12425
47	ON	Okinawa		10082

We have used hierarchical administrative subdivision codes for the prefectures (http://www.statoids.com/ujp.html).

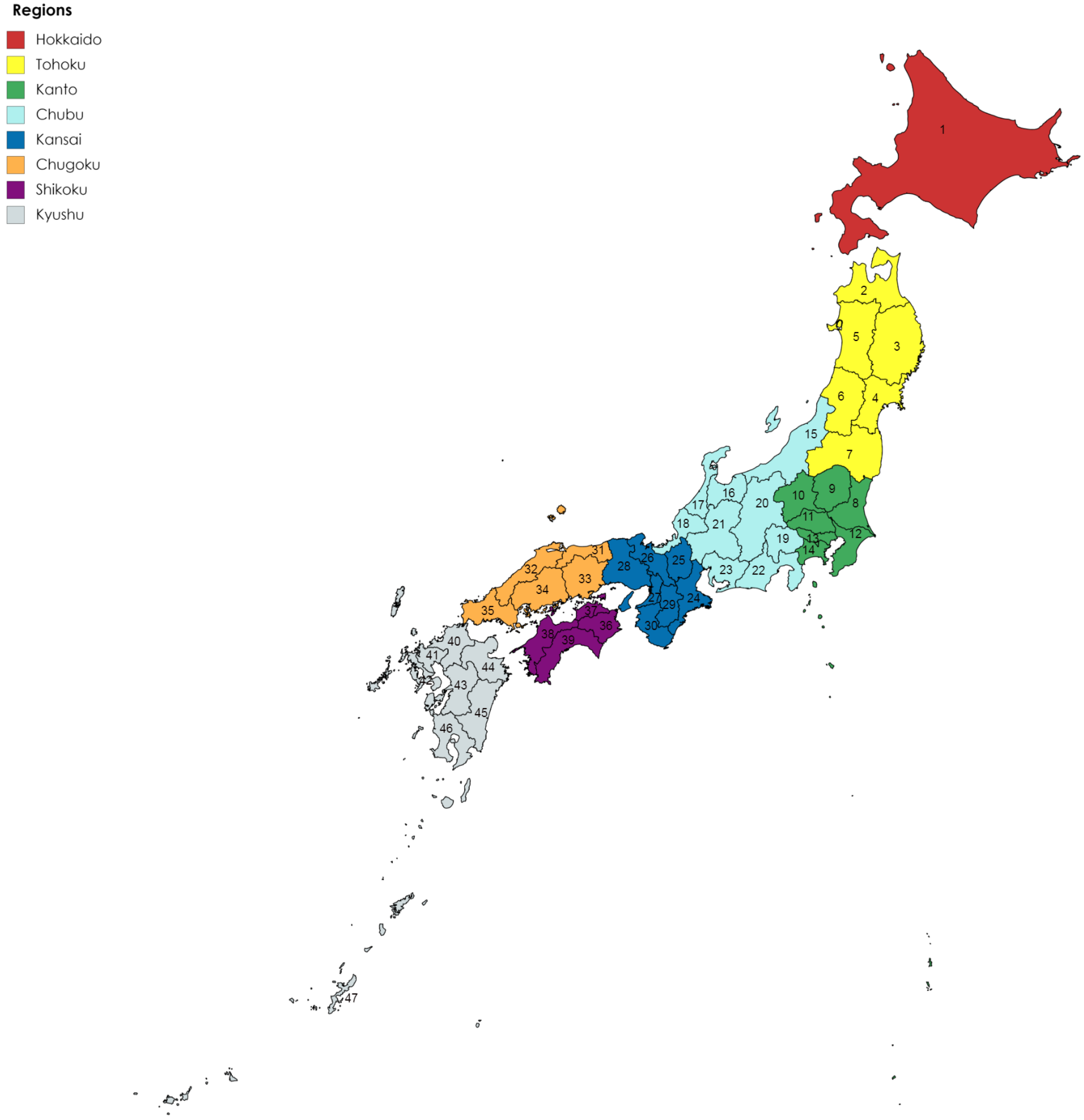

S1 Fig. **The 8 regions and 47 prefectures in Japan.** The numbers represent IDs of the prefectures as given in S1 Table. The map is created using https://mapchart.net/japan.html.

S2 Table. **Industrial sectors and divisions**

ID	Sectors	Divisions
1	Agriculture	Agriculture & Forestry
2	Forestry	
3	Fisheries, except Aquaculture	Fisheries
4	Aquaculture	
5	Mining and quarrying of stone	Mining and quarrying of stone
6	Construction work, general including public and private construction work	
7	Construction work by specialist contractor,except equipment installation work	Construction
8	Equipment installation work	
9	Manufacture of food	
10	Manufacture of beverages	
11	Manufacture of textile products	
12	Manufacture of lumber and wood products, except furniture	
13	Manufacture of furniture and fixtures	
14	Manufacture of pulp, paper and paper products	
15	Printing and allied industries	
16	Manufacture of chemical and allied product	
17	Manufacture of plastic products, except otherwise classified	
18	Manufacture of rubber products	
19	Manufacture of leather tanning,leather products and fur skins	
20	Manufacture of ceramic, stone and clay products	Manufacturing
21	Manufacture of iron and steel	
22	Manufacture of non-ferrous metals and products	
23	Manufacture of fabricated metal products	
24	Manufacture of general-purpose machinery	
25	Manufacture of production machinery	
26	Manufacture of business oriented machinery	
27	Electronic parts, devices and electronic circuits	
28	Manufacture of electrical machinery, equipment and supplies	
29	Manufacture of information and communication electronics equipment	
30	Manufacture of transportation equipment	
31	Miscellaneous manufacturing industries	
32	Production, transmission and distribution of electricity	
33	Production and distribution of gas	Electricity, Gas, Heat & Water
34	Heat supply	
35	Collection, purification and distribution of water and sewage collection, processing and disposal	
36	Communications	
37	Broadcasting	Information & Communications
38	Information services	
39	Video picture information, sound information, character information production and distribution	
40	Railway transport	
41	Road Passenger transport	
42	Road freight transport	
43	Water transport	Transport & Postal service
44	Air transport	
45	Warehousing	
46	Services incidental to transport	
47	Postal services, including mail delivery	

S3 Table. **Industrial sectors and divisions**

ID	Sectors	Divisions
48	Wholesale trade, general merchandise	
49	Wholesale trade (textile and apparel)	
50	Wholesale trade (food and beverages)	
51	Wholesale trade (building materials, minerals and metals, etc.)	
52	Wholesale trade (machinery and equipment)	
53	Miscellaneous Wholesale trade	Wholesale & Retail trade
54	Retail trade, general merchandise	
55	Retail trade (woven fabrics, apparel, apparel accessories and notions)	
56	Retail trade (food and beverage)	
57	Retail trade (machinery and equipment)	
58	Miscellaneous retail trade	
59	Nonstore retailers	
60	Banking	
61	Financial institutions for cooperative organizations	
62	Non-deposit money corporations, including lending and credit card business	Finance & Insurance
63	Financial auxiliaries	
64	Insurance institutions, including insurance agents brokers and services	
65	Real estate agencies	
66	Real estate lessors and managers	Real estate & Goods rental
67	Goods rental and leasing	
68	Scientific and development research institutes	Scientific research & Technical services
69	Technical services, N.E.C.	
70	Accommodations	
71	Eating and drinking places	Accommodations & Eating services
72	Food take out and delivery services	
73	Laundry, beauty, and bath services	
74	Miscellaneous living-related and personal services	Living-related and personal services
75	Services for amusement and recreation	
76	School education	Education & Learning support
77	Miscellaneous education, learning support	
78	Medical and other health services	
79	Public health and hygiene	Medical health care and welfare
80	Social insurance, social welfare and care services	
81	Cooperative associations, N.E.C	Cooperative associations
82	Waste disposal business	
83	Automobile maintenance services	
84	Machine, etc. repair services, except otherwise classified	
85	Employment and worker dispatching services	Service, N.E.C.
86	Miscellaneous business services	
87	Political, business and cultural organizations	
88	Religion	
89	Miscellaneous services	
90	National government services	Government services
91	Local government services	

S4 Table. **Ranking of the prefectures using Economic Complexity Index (ECI) and Fitness**

Rank	ECI	Fitness
1	Tokyo	Tokyo
2	Aichi	Osaka
3	Osaka	Aichi
4	Kanagawa	Kanagawa
5	Hyogo	Hyogo
6	Fukuoka	Chiba
7	Saitama	Ibaraki
8	Kyoto	Saitama
9	Toyama	Fukuoka
10	Hiroshima	Okinawa
11	Chiba	Toyama
12	Okinawa	Mie
13	Shizuoka	Kyoto
14	Okayama	Shizuoka
15	Ishikawa	Hokkaido
16	Fukui	Oita
17	Kagawa	Okayama
18	Nara	Gifu
19	Ehime	Ehime
20	Yamaguchi	Hiroshima
21	Mie	Kagawa
22	Gifu	Nara
23	Oita	Tochigi
24	Tochigi	Yamaguchi
25	Shiga	Tokushima
26	Ibaraki	Ishikawa
27	Wakayama	Fukushima
28	Tokushima	Wakayama
29	Hokkaido	Niigata
30	Fukushima	Saga
31	Yamanashi	Shiga
32	Nagano	Kagoshima
33	Gunma	Yamanashi
34	Miyagi	Aomori
35	Niigata	Fukui
36	Shimane	Shimane
37	Yamagata	Tottori
38	Saga	Yamagata
39	Tottori	Nagasaki
40	Nagasaki	Nagano
41	Aomori	Gunma
42	Kagoshima	Kumamoto
43	Kumamoto	Miyazaki
44	Miyazaki	Miyagi
45	Akita	Akita
46	Kochi	Kochi
47	Iwate	Iwate

S5 Table. **Top and bottom 10 industrial sectors ranked by Product Complexity Index (PCI) and Complexity.**

Rank	PCI	Complexity
1	Video picture, sound, character information production and distribution	Video picture, sound, character information production and distribution
2	Wholesale trade, general merchandise	Communications
3	Communications	Insurance institutions, including insurance agensts, brokers and services
4	Insurance institutions, including insurance agensts, brokers and services	Wholesale trade, general merchandise
5	Railway Transport	Postal services including mail delivery
6	Postal services including mail delivery	National government services
7	Wholesale trade (Building materials, Minerals and Metals, etc)	Real estate lessors and managers
8	Manufacture of iron and steel	Railway Transport
9	National government services	Financial auxiliaries
10	Real estate lessors and managers	Information Services
82	Manufacture of food	Forestry
83	Forestry	Miscellaneous retail trade
84	Wholesale trade (food and beverages)	Medical and other health services
85	Manufacture of lumber and wood products except furniture	Construction work, general including public and private
86	Services for amusement and recreation	Road passerger transport
87	Accommodations	Automobile maintenance services
88	Cooperative assocations, N.E.C.	Retail trade (Machinery and equipment)
89	Agriculture	Waste disposal business
90	Fisheries, except aquaculture	Construction work by specialist contractor, except equipment installation work
91	Miscellaneous services	Local government services

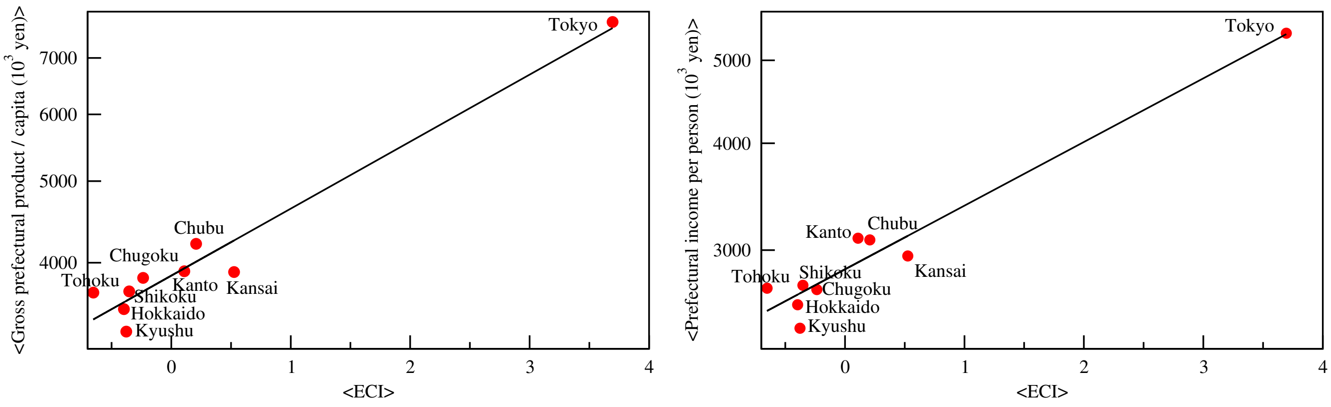

S2 Fig. **The variation of average prefectural economic coplexity index $< ECI >$ with (a) average gross prefec-tural product per capita and (b) average prefectural income per person in eight regions and Tokyo.** The straight line in both plots represnts the best exponential fit to the data, indicating the expected values of the average per-capita gross prefectural product and average prefectural income per person. The average is taken over all the prefectures of a region.

THE AVERAGE PREFECTURAL ECONOMIC COMPLEXITY OF REGIONS IN JAPAN

To relate the observed ECI with the regions in Japan, we have measured the average refectural economic complexity $< ECI >$ of each regions in Japan. The average prefectural economic complexity $< ECI >$ is found to be strongly correlated with average gross prefectural product per capita (Pearson's product-moment correlation $r = 0.979$ and $p{-}value = 4.27{\times}10^{-06}$) and average prefectural income per person (Pearson's product-moment correlation $r = 0.982$ and $p - value = 2.23 \times 10^{-06}$) in the eight regions and Tokyo. As can be seen from the S2 Fig, Tohuku region has the least average prefectural economic complexity. However, this region is performing well comapared to its ECI. It also shows that the Kyushu region has low ECI and this region is not performing well.
