# Supplementary material for: Economic complexity of prefectures in Japan
Source: PLoS One. 2020 Aug 27;15(8):e0238017. doi: 10.1371/journal.pone.0238017 (PMC7451641; doi:10.1371/journal.pone.0238017)
Supplement: Attachment — Submitted filename: PONE-D-20-04110_referee_report.pdf [file pone.0238017.s002.pdf]

Referee report for the paper Economic complexity of prefectures in Japan

Thank you for allowing me to review this interesting paper, I recommend its publication but only after some comments which I enumerate below are dealt with:

1) The authors should justify their clustering method as there are other methods like stochastic block models or other recent applications like for bank-firms bipartite networks which can be used for bipartite networks
2) The data set is fantastic and the analysis interesting. However, a study using more granularity would be very welcomed. The authors thought about a prefecture-firm study, for example. In this way the complexity of prefectures would be based on individual firms, as well as the complexity of firms (maybe less interesting).
3) Is there a specific reason for the authors to use the unweighted version of the bipartite network? I think that the weighted version can be quite useful in order to put monetary value to the metrics, even for the projections the monetary value of the similarity can be quite useful as well as a possible centrality study.
