# Supplementary material for: Economic complexity of prefectures in Japan
Source: PLoS One. 2020 Aug 27;15(8):e0238017. doi: 10.1371/journal.pone.0238017 (PMC7451641; doi:10.1371/journal.pone.0238017)
Supplement: Attachment — Submitted filename: Response_to_Reviewers.pdf [file pone.0238017.s003.pdf]

Detailed responses to the comments by the Referees on manuscript PONE-D-20-04110:

We thank the Referees for their encouraging and thoughtful remarks as well as for their helpful suggestions. We reproduced all of their points below (in italics) together with our responses (in non-italics) to all points of criticism and suggestions.

The changes in the revision result mostly from their suggestions and are therefore listed as well. The changes have been listed at the end.

Journal Requirements:

When submitting your revision, we need you to address these additional requirements:

1. Please ensure that your manuscript meets PLOS ONE's style requirements, including those for file naming. The PLOS ONE style templates can be found at http://www.plosone.org/attachments/PLOSOne_formatting_sample_main_body.pdf and http://www.plosone.org/attachments/PLOSOne_formatting_sample_title_authors_affiliations.pdf

We have revised the manuscript to meet PLOS ONE's style requirements.

2. We note that you have indicated that data from this study are available upon request. PLOS only allows data to be available upon request if there are legal or ethical restrictions on sharing data publicly. For more information on unacceptable data access restrictions, please see http://journals.plos.org/plosone/s/data-availability#loc-unacceptable-data-access-restrictions.

In your revised cover letter, please address the following prompts:

a) If there are ethical or legal restrictions on sharing a de-identified data set, please explain them in detail (e.g., data contain potentially sensitive information, data are owned by a third-party organization, etc.) and who has imposed them (e.g., an ethics committee). Please also provide contact information for a data access committee, ethics committee, or other institutional body to which data requests may be sent.

The data for bipartite network is based on a survey done by Tokyo Shoko Research (TSR), one of the leading credit research agencies in Tokyo, supplied to us through the Research Institute of Economy, Trade and Industry (RIETI).
The data are not in the public domain, but are commercially available. Information on

data access is given below.
Provider: Tokyo Shoko Research, Ltd.
JA Bldg., 1-3-1 Otemachi, Chiyoda-ku, Tokyo 100-6810, JAPAN
Tel: +81 (0)3-6910-3142
Fax: +81 (0)3-5221-0712
Web: http://www.tsr-net.co.jp/
Database: TSR Company Profile Data File
Description:
TSR Company Profile Data File is standard data sets that have been provided for many years in local market. The data is based on TSR's reporters' site visit interviews which is our most frequent data source.
TSR offers various services/ data in accordance with customers' demand, such as TSR Report, TSR Company Profile Data File, financial statement data, Internet Service "tsrvan2", viability scores, etc.
We collected the gross prefectural product data, prefectural population data and prefectural income per person data for the year 2015 from the Japanese government statistical portal site (https://www.e-stat.go.jp), which are in the public domain.

b) If there are no restrictions, please upload the minimal anonymized data set necessary to replicate your study findings as either Supporting Information files or to a stable, public repository and provide us with the relevant URLs, DOIs, or accession numbers. For a list of acceptable repositories, please see http://journals.plos.org/plosone/s/data-availability#loc-recommended-repositories.

As we explain above, the data are not in the public domain but are commercially available. Therefore, we cannot upload the minimal anonymized data set.

We will update your Data Availability statement on your behalf to reflect the information you provide.

3. Please include captions for your Supporting Information files at the end of your manuscript, and update any in-text citations to match accordingly. Please see our Supporting Information guidelines for more information: http://journals.plos.org/plosone/s/supporting-information.

We have revised it accordingly.

Reviewer #1:

Reviewer #1: In their articles, the Authors analyze the economic complexity of the Japan industrial sector, with the aim of relating the healthy of the economic system with the geographical division of the considered region. In particular, they analyze the different

prefectures that are present in Japan, firstly considering how similar are the industrial sectors that can be found in each of the prefectures. Then, they apply a series of published methods that are ad-hoc adapted for their case study to evaluate the economic health of each of the prefectures. The analysis is deeply done, and it can be followed even from readers, like me, that are not expert in this specific research field (a thing that is valuable for a broad impact journal like PlosOne).

We are pleased that the reviewer found that "The analysis is deeply done, and it can be followed even from readers, like me, that are not expert in this specific research field (a thing that is valuable for a broad impact journal like PlosOne)."

I have three minor criticisms/suggestions, that in my opinion would greatly improve the paper:

1. To allow an easier understanding of the results even to not Japanese people, I suggest the Authors to add at list a figure in which there is the Japan geographical map divided into the different prefectures. The best, would be to present each of the obtained results suitably color coding a map: this would be very easy, for example, for Figs 1 and 2 (you just have to color the founded clusters of the tree and put the figure side of the tree...). A step in this direction would be very interesting also for the other figures, even if less obvious.

We thank the reviewer for this excellent idea. We have added a map of Japan in the Appendix with the same color code as used for the regions in Fig 1.

Changes made in the manuscript:
At Fig 1 caption: "The eight regions of Japan are shown in a map using the same color code in Fig. S1 of Appendix S1."

Changes in Appendix S1:
We have added a map of Japan in Fig S1 to show the 8 regions and the 47 prefectures in Japan.

2. While the study is based on the geographical unit of the prefecture, it would be interesting to give an insight on the impact of the regional division. How the obtained results relate with the divisions in regions of the prefectures?

To relate the obtained results with the divisions in region we have added a section "The average prefectural economic complexity of regions in Japan" in the Appendix S1.

Changes made in the manuscript:
At line 237-239: "An interpretation of the above results for the regions in Japan is given in the section "the average prefectural economic complexity of regions in Japan" of Appendix S1."

Changes made in Appendix S1:
We have added a section ``the average prefectural economic complexity of regions in Japan"
We have added Fig S2.

3. Finally, I think that the work the authors presented at the Complex Network conference that has exactly the same title should be properly referenced in the paper.

We presented some of the very preliminary results with the same title at the Complex Network conference 2019. A short abstract was only published in the "Book of Abstracts". We feel this citation may not be necessary here.

Reviewer #2:

The authors apply the economic complexity methodology to the bipartite network of economic sectors and Japanese prefectures. Even if I am sympathetic with respect to the methodology, I do not think that the manuscript can be published in the present form. The authors could try to deeply revise it, trying to add economical and or theoretical investigations.
The main problem is the scientific contribution of this manuscript.
It is stated in the abstract that "studies on economic complexity at the regional level are lacking", quite in contradiction with lines 32-33 "economic complexity has been studied at the regional level for China [12], Brazil [10], the US and the UK [5]", and I could add Mexico [1], Italy [2], Spain [3], and even Australia [4]. All these studies follow more or less the same route: first, a database is obtained with the export structure (i.e., different products) of each sub-national entity; then some normalization is performed (RCA); then the ECI or the Fitness or the Product Space algorithm is applied.
I believe that publishing the same exercise with different data is not very interesting from a scientific point of view. This analysis can be of some utility for Japanese policymakers, but does not add much to these known methodologies and also the economic meaning is not much discussed, for instance, with respect to more standard economical approaches. In conclusion, I find the data interesting but I think the authors should make a large effort to provide some better contribution to the literature.

Most of these regional complexity studies are done at very coarse grain level. In case of China, the analysis is performed for 31 provinces with 2690 firms, which is a tiny fraction of all Chinese firms. The complexity analysis is performed at states level for Brazil, Mexico and Australia. Moreover, the aims of some of these studies are very different from our analysis. For example, the regional complexity analysis of US and UK has been used as an example for the interpretation of ECI. What is very different in our study is that it concerns supply-chain in which regions and industrial sectors are studied, so one is looking at process of value added starting from a giant network of firms and by aggregating as a binary bipartite network. Here we performed a detailed complexity analysis of the prefectures in Japan using ECI and fitness complexity algorithm. Furthermore, our analysis revels the similarity among prefectures and among industrial sectors in Japan. The data of our study covers a large fraction of firms in Japan. Japan has been one of the most diversified country in the sense of the products. Therefore, it is important to reveal that whether such diversity comes from regional structures.

We thank the referee for altering us to these papers. We have now incorporated these references in the manuscript.

Changes made in the manuscript:

At the abstract: “Although there have been extensive studies on countries' economic complexity using international export data, studies on economic complexity at the regional level are relatively less studied.”

At line 32-40: “Recently, economic complexity has been studied at the regional level for China [12], Brazil [10], Mexico [13], Italy [14], Spain [15], Australia [16], the US and the UK [5]. Most of these regional complexity studies are done at very coarse grain level. In case of China, the analysis is performed for 31 provinces with 2690 firms, which is a tiny fraction of all Chinese firms. The complexity analysis is performed at states level for Brazil, Mexico and Australia. The difference in our study is that it concerns supply-chain in which prefectures and industrial sectors are studied. We are looking at process of value added starting from a giant network of firms and by aggregating as a binary bipartite network of prefectures and industrial sectors.”

At line 46-48: “Japan has been one of the most diversified country in the sense of the products. Therefore, it is important to reveal that whether such diversity comes from regional structures.”

At line 78-79: “These firms constitute a giant weakly connected component in the Japanese production network [21].”

Moreover, other important methodological issues are present:
- Some sectors are excluded from the analysis in a quite arbitrary way (as stated, because they are only linked to Tokyo, and this results in zero fitness for the other prefectures). Whether this is a data or a methodological problem, it should be clearly stated and discussed.

In the manuscript, at line 88-89, we have already mentioned that “the inclusion of these sectors in our analysis results in the largest value for the fitness of Tokyo, and the fitness of other prefectures become zero.”

- The economic complexity methodology is usually applied to export data. Here RCA is computed from annual sales, which include internal and external production in a highly biased way. Both the use of sales instead of export and RCA should be motivated, possibly with some references.

Now we have included our motivation for the use of sales instead of export with references.

Changes made in the manuscript:
At line 92-100: “The Revealed Comparative Advantage (RCA)) [Balassa 1965] is frequently used as a quantitative criterion to evaluate the relative dominance of a country, in the export of

certain products by comparing it with the average export of those products. Recently, RCA has been measured from the ratio between the actual number of firms from an industry in a province and the average number of firms from that industry in that province [Gao 2018]. Mealy et al. constructed a binary region-industry matrix based on the number of people employed in an industry in a region [Mealy 2019]. Here, we use annual sales of industrial sector s in prefecture p to measure the RCA."

- From Figures 4 and 6 it emerges that all the correlations are driven by Tokyo. What happens if it is removed?

Our analysis shows that the Tokyo has highest economic complexity or fitness. Therefore, it plays an important role. If we remove the data for Tokyo in our correlation measures we find the following results:
The Pearson's product-moment correlation between ECI and GPP per Capita is $r = 0.454$ and $p\text{-value} = 0.001$.
The Pearson's product-moment correlation between ECI and Prefectural income per person $r= 0.500$ and $p\text{-value} = 0.0004$.

The Pearson's product-moment correlation between fitness and GPP per Capita is $r = 0.241$ and $p\text{-value} = 0.107$.
The Pearson's product-moment correlation between fitness and Prefectural income per person $r= 0.243$ and $p\text{-value} = 0.104$.

The above results show that the correlations measures are not solely driven by Tokyo (at least in case of ECI and the macro-economic indicators).

Minor issues:
- Lines 101-102 are inaccurate. k_{p,N} is not the diversity of prefecture, as incorrectly stated, but the ECI. The diversity is k0. The same applies to sectors.

We thank the reviewer for pointing it out. We have now modified the line in the manuscript.

Changes made in the manuscript
At line 122-124: "The economic complexity index (ECI) of prefectures and product complexity index (PCI) of industrial sectors can be calculated using the following iterative equation:"

- The mathematical derivation in lines 104-125 is well known in the literature (ref. [7] and [24] of the paper), so it is quite useless.
- Also, the (quite elementary) similarity measures for both prefectures (pag.5) and sectors (page 6) is from [7].

We agree with the reviewer that the mathematical derivation in lines 104-125 is well known in the literature and also the similarity measures are very basic. However, we believe it will be

helpful for the readers to follow the article in a compact way. So we keep it as it is.

- On lines 188-189 it is stated that "k p,0 and k p,1 are slightly negatively correlated (Pearson correlation coefficient r = −0.230 and p-value = 0.119)", which I find rather surprising. These iterative methods are supposed to converge to some "real value" of the economic complexity, providing better and better assessments as the iteration procedure goes on, so I would expect them to be highly correlated.

We found the correlation low because these are initial iterations, however we have observed the correlation becomes strong at the subsequent iterations. The Pearson correlation coefficient between $k_{p,1}$ and $k_{p,2}$ is found $r = -0.630$ and $p\text{-value} = 2.05 \times 10^{-6}$.

[1] Chávez, J. C., Mosqueda, M. T., & Gómez-Zaldívar, M. (2017). Economic complexity and regional growth performance: Evidence from the Mexican Economy. Review of Regional Studies, 47(2), 201-219.
[2] Basile, R., Cicerone, G., & Iapadre, L. (2019). Economic complexity and regional labor productivity distribution: evidence from Italy.
[3] Balsalobre, S. J. P., Verduras, C. L., & Lanchas, J. D. (2017). Measuring the Economic Complexity at the sub-national level using international and interregional trade.
[4] Reynolds, C., Agrawal, M., Lee, I., Zhan, C., Li, J., Taylor, P., ... & Roos, G. (2018). A sub-national economic complexity analysis of Australia's states and territories. Regional Studies, 52(5), 715-726.

Reviewer #3:

The paper is technically sound and the database is fantastic.
The statistical analysis is also adequate but more could be done with such a detailed database
The paper is also well written in English
The authors have made clear where the data was got from but I am not sure if they can share the detailed information on the firms. Could you please clarify this issue?

We cannot share the detailed information on firms as the data are not in the public domain but are commercially available.

The data for bipartite network is based on a survey done by Tokyo Shoko Research (TSR), one of the leading credit research agencies in Tokyo, supplied to us through the Research Institute of Economy, Trade and Industry (RIETI).
The data are not in the public domain, but are commercially available. Information on data access is given below.
Provider: Tokyo Shoko Research, Ltd.
JA Bldg., 1-3-1 Otemachi, Chiyoda-ku, Tokyo 100-6810, JAPAN
Tel: +81 (0)3-6910-3142
Fax: +81 (0)3-5221-0712

Web: http://www.tsr-net.co.jp/
Database: TSR Company Profile Data File
Description:
TSR Company Profile Data File is standard data sets that have been provided for many years in local market. The data is based on TSR's reporters' site visit interviews which is our most frequent data source.
TSR offers various services/ data in accordance with customers' demand, such as TSR Report, TSR Company Profile Data File, financial statement data, Internet Service "tsrvan2", viability scores, etc.
We collected the gross prefectural product data, prefectural population data and prefectural income per person data for the year 2015 from the Japanese government statistical portal site (https://www.e-stat.go.jp), which are in the public domain.

Thank you for allowing me to review this interesting paper, I recommend its publication but only after some comments which I enumerate below are dealt with:
1) The authors should justify their clustering method as there are other methods like stochastic block models or other recent applications like for bank-firms bipartite networks which can be used for bipartite networks

We agree with the reviewer that there are many methods for clustering analysis. However, most of them give very similar results. Moreover, our network is similar to a correlation based network. The minimal spanning tree is a widely used technique for clustering analysis for this type of network. Methods like stochastic block models are mostly used for sparse networks.

2) The data set is fantastic and the analysis interesting. However, a study using more granularity would be very welcomed. The authors thought about a prefecture-firm study, for example. In this way the complexity of prefectures would be based on individual firms, as well as the complexity of firms (maybe less interesting).

Prefecture-firm network may not be suitable for this study. As each firm belongs to a single prefecture only. The degree of each firm will be one in that case, indicating a homogeneity.

3) Is there a specific reason for the authors to use the unweighted version of the bipartite network? I think that the weighted version can be quite useful in order to put monetary value to the metrics, even for the projections the monetary value of the similarity can be quite useful as well as a possible centrality study.

The algorithm that measures economic complexity index and product complexity index is applied on a binary bipartite matrix [PNAS 106(26),10570 (2009)]. This is the reason we have used the unweighted version of the bipartite network.

.

To conclude, we again thank all the reviewers for providing us with the opportunity to clarify these issues. We have updated our manuscript (as detailed in the attached "List of Changes to the Manuscript") according the points discussed above. We hope that the changes we have made to the manuscript address the reviewers' concerns.

<u>List of Changes to the Manuscript</u>

1. We have updated affiliations in the manuscript.

2. At the abstract: "Although there have been extensive studies on countries' economic complexity using international export data, studies on economic complexity at the regional level are relatively less studied."

3. At line 32-40: "Recently, economic complexity has been studied at the regional level for China [12], Brazil [10], Mexico [13], Italy [14], Spain [15], Australia [16], the US and the UK [5]. Most of these regional complexity studies are done at very coarse grain level. In case of China, the analysis is performed for 31 provinces with 2690 firms, which is a tiny fraction of all Chinese firms. The complexity analysis is performed at states level for Brazil, Mexico and Australia. The difference in our study is that it concerns supply-chain in which prefectures and industrial sectors are studied. We are looking at process of value added starting from a giant network of firms and by aggregating as a binary bipartite network of prefectures and industrial sectors."

4. At line 46-48: "Japan has been one of the most diversified country in the sense of the products. Therefore, it is important to reveal that whether such diversity comes from regional structures."

5. At line 78-79: "These firms constitute a giant weakly connected component in the Japanese production network [21]."

6. At line 92-100: "The Revealed Comparative Advantage (RCA)) [Balassa 1965] is frequently used as a quantitative criterion to evaluate the relative dominance of a country, in the export of certain products by comparing it with the average export of those products. Recently, RCA has been measured from the ratio between the actual number of firms from an industry in a province and the average number of firms from that industry in that province [Gao 2018]. Mealy et al. constructed a binary region-industry matrix based on the number of people employed in an industry in a region [Mealy 2019]. Here, we use annual sales of industrial sector s in prefecture p to measure the RCA."

7. At line 122-124: "The economic complexity index (ECI) of prefectures and product complexity index (PCI) of industrial sectors can be calculated using the following iterative equation:"

8. At Fig 1 caption: "The eight regions of Japan are shown in a map using the same color code in

Fig. S1 of Appendix S1."

9. At line 237-239: "An interpretation of the above results for the regions in Japan is given in the section "the average prefectural economic complexity of regions in Japan" of Appendix S1."

10. At the Appendix S1: We have added a map of Japan in Fig S1 to show the 8 regions and the 47 prefectures in Japan.

11. At the Appendix S1: We have added a section ``the average prefectural economic complexity of regions in Japan" and Fig S2.
