# Supplementary material for: Economic complexity of prefectures in Japan
Source: PLoS One. 2020 Aug 27;15(8):e0238017. doi: 10.1371/journal.pone.0238017 (PMC7451641; doi:10.1371/journal.pone.0238017)
Supplement: Attachment — Submitted filename: Response_to_Reviewers.pdf [file pone.0238017.s004.pdf]

Detailed responses to the comments by the Referees on manuscript PONE-D-20-04110R1:

We thank the Referees for their encouraging and thoughtful remarks as well as for their helpful suggestions. We reproduced all of their points below (in italics) together with our responses (in non-italics) to all points of criticism and suggestions.

The changes in the revision result mostly from their suggestions and are therefore listed as well.

Reviewers' comments:

Reviewer's Responses to Questions

Comments to the Author

1. If the authors have adequately addressed your comments raised in a previous round of review and you feel that this manuscript is now acceptable for publication, you may indicate that here to bypass the "Comments to the Author" section, enter your conflict of interest statement in the "Confidential to Editor" section, and submit your "Accept" recommendation.

Reviewer #1: All comments have been addressed

Reviewer #3: (No Response)

2. Is the manuscript technically sound, and do the data support the conclusions?

The manuscript must describe a technically sound piece of scientific research with data that supports the conclusions. Experiments must have been conducted rigorously, with appropriate controls, replication, and sample sizes. The conclusions must be drawn appropriately based on the data presented.

Reviewer #1: Yes

Reviewer #3: Yes

3. Has the statistical analysis been performed appropriately and rigorously?

Reviewer #1: Yes

Reviewer #3: Yes

4. Have the authors made all data underlying the findings in their manuscript fully available?

The PLOS Data policy requires authors to make all data underlying the findings described in their manuscript fully available without restriction, with rare exception (please refer to the Data Availability Statement in the manuscript PDF file). The data should be provided as part of the manuscript or its supporting information, or deposited to a public repository. For example, in addition to summary statistics, the data points behind means, medians and variance measures should be available. If there are restrictions on publicly sharing data—e.g. participant privacy or use of data from a third party—those must be specified.

Reviewer #1: No

Reviewer #3: Yes

We have already provided a data availability statement which Plos one has approved by email communication with us on July 9, 2020. We share the statement below for your reference.

‘’The data for bipartite network is based on a survey done by Tokyo Shoko Research (http://www.tsr-net.co.jp/), one of the leading credit research agencies in Tokyo, and is supplied through the Research Institute of Economy, Trade and Industry. The data are not in the public domain, but are commercially available. Data access requests for the TSR Company Profile Data File can be directed to the Tokyo Shoko Research, Ltd. (contact via telephone: +81 (0)3-6910-3142, or via fax: +81 (0)3-5221-0712). Therefore, the data can be accessed by others in the same manner by which the authors obtained them.’’

5. Is the manuscript presented in an intelligible fashion and written in standard English?

PLOS ONE does not copyedit accepted manuscripts, so the language in submitted articles must be clear, correct, and unambiguous. Any typographical or grammatical errors should be corrected at revision, so please note any specific errors here.

Reviewer #1: Yes

Reviewer #3: Yes

6. Review Comments to the Author

Reviewer #1: I'm completely satisfied with the Authors' paper revision, even if most of my comments have been answered in the SI. In my opinion, given also the Authors' reply, some figure should be moved in the main text, but I leave the Authors do what they think is the best.

We are pleased to know that our responses were helpful to address the reviewer's concerns. We kept these figures in the SI such that readers do not divert from the main context.

Reviewer #3: I made a question in my previous revision but I am not satisfied with the answer. The authors should elaborate a lot more in their response and include it in the paper. I am referring to the question and reply below:

3) Is there a specific reason for the authors to use the unweighted version of the bipartite network? I think that the weighted version can be quite useful in order to put monetary value to the metrics, even for the projections the monetary value of the similarity can be quite useful as well as a possible centrality study.

The algorithm that measures economic complexity index and product complexity index is applied on a binary bipartite matrix [PNAS 106(26),10570 (2009)]. This is the reason we have used the unweighted version of the bipartite network.

We understand the reviewer's concern but the original economic complexity is defined as binary networks. Since we can compare our findings with results of the original economic complexity, we employ the original economic complexity. In addition, the expansion of the definition to the weighted networks can be another work and is beyond our scope.

Changes made in the manuscript:
At line 278-280: "We have studied economic complexity of prefectures in Japan based on the binary bipartite matrix. In the future, it will be interesting to see if one gets more valuable insights using a weighted matrix."

To conclude, we again thank all the reviewers for providing us with the opportunity to clarify these issues. We have updated our manuscript according to the points discussed above. We hope that the changes we have made to the manuscript address the reviewers' concerns.
